# A global analysis of population differentiation at the major histocompatibility complex genes across tetrapods

**DOI:** 10.1098/rspb.2026.1096

**Published:** 2026-10-14

**Authors:** Piotr Minias, Amelia Chyb, Tomasz Gaczorek, Wiesław Babik

**Affiliations:** ^1^ Department of Biodiversity Studies and Bioeducation, University of Lodz, Łódź, Poland; ^2^ Laboratory of Bioinformatics and Genome Biology, Jagiellonian University, Kraków, Poland; ^3^ Institute of Environmental Sciences, Jagiellonian University, Kraków, Poland

**Keywords:** gene copy number, local adaptation, major histocompatibility complex (MHC), pathogen-driven selection, population differentiation, comparative analysis

## Abstract

Major histocompatibility complex (MHC) genes are central to pathogen recognition by the adaptive immune system of jawed vertebrates, with MHC classes I and II (henceforth referred to as MHC-I and MHC-II, respectively) primarily mediating responses to intracellular and extracellular pathogens, respectively. Here, we tested the hypothesis that MHC-II genes show stronger population differentiation, reflecting spatially heterogeneous selection by extracellular pathogens. We compiled a global dataset comprising >3500 pairwise estimates of MHC differentiation across approximately 120 tetrapod species. Phylogenetically informed analyses revealed consistently stronger differentiation at MHC-II than at MHC-I genes, supporting stronger local adaptation to spatially variable selection by extracellular pathogens. Isolation by distance contributed broadly to MHC differentiation, indicating that balancing selection is insufficient to homogenize MHC variation over large geographical scales. Differentiation varied markedly among tetrapod lineages, reflecting the differences in dispersal capacity. We further showed that differentiation was modulated by the genomic architecture of the MHC region, and our theoretical simulations demonstrated that variation in MHC gene copy number can have contrasting effects on population differentiation, depending on the strength of balancing selection acting on each MHC class. Together, these findings elucidate the global patterns of immunogenetic differentiation and advance the mechanistic understanding of how extraordinary MHC variation is structured across space.

## 1. Introduction

 Hosts and pathogens form dynamic, coevolving systems, governed by arms-race processes, in which the host immune system evolves to recognize, combat or tolerate pathogens, while pathogens evolve to evade immune defences and establish infection [1]. Host–pathogen coevolution is broadly explained by the Red Queen hypothesis, which has received widespread theoretical and empirical support across diverse biological systems [2,3,4]. As pathogens adapt to common host genotypes, novel or rare immune variants gain a selective advantage, driving reciprocal selection for novel or rare escape variants in pathogens [5]. Red Queen processes result in negative frequency-dependent selection, which is particularly well documented at the level of host immune pathways involved in pathogen recognition [6,7]. However, as both host and pathogen populations are often geographically and ecologically structured, coevolutionary dynamics can vary across space and environment [8,9]. Consequently, the fitness of immune variants may fluctuate not only over time but also among locations, microhabitats or ecological niches [10,11]. These processes promote local adaptation, whereby host defences become adapted to local pathogen communities and environmental conditions, leading to geographical differentiation in immune genes [12].

Of the two branches of vertebrate immunity, the innate branch relies on germline-encoded receptors and effectors that recognize and respond primarily to conserved pathogen-associated molecular patterns [13]. As a result, innate immunity genes tend to evolve relatively slowly and respond poorly to rapid fluctuations in parasite-mediated selection [14]. Although coevolutionary dynamics involving vertebrate innate immune genes and pathogens have been suggested [15], such cases appear idiosyncratic and episodic, limiting the scope for broad generalization [16]. By contrast, the adaptive immune system relies on the somatic generation of an extraordinary diversity of lymphocyte receptors and antibodies, enabling responses even to rapidly evolving pathogen antigens. Triggering an effective immune response requires the presentation of pathogen-derived antigenic peptides to T cells carrying diverse receptors. This process is mediated by germline-encoded major histocompatibility complex (MHC) molecules that often coevolve with pathogens. Each MHC molecule presents only a limited spectrum of antigenic peptides, primarily determined by the amino acid sequence of its peptide-binding groove [17]. However, MHC alleles can differ substantially in the breadth of peptide repertoires they are capable of presenting, which has led to the concept of specialist and generalist alleles [18]. Two classes of MHC molecules, encoded by class I and class II genes (henceforth referred to as MHC-I and MHC-II, respectively), mediate the presentation of antigens derived from intracellular and extracellular pathogens, respectively (but see [19] for MHC-I cross-presentation).

The MHC represents the most polymorphic genes in jawed vertebrates. In humans, more than 43 000 distinct MHC allelic variants have been described to date [20], with an expected 3 million rare variants per locus [21]. Hundreds or thousands of variants have also been identified in some wild vertebrate species [22,23]. This remarkable hyper-polymorphism is maintained by balancing selection operating through a combination of several mechanisms, including negative frequency-dependent selection (NFS), fluctuating selection and heterozygote advantage [24,25]. However, MHC polymorphism at the individual level is primarily determined by the number of MHC gene copies (i.e. loci). On the one hand, a higher number of MHC gene copies increases the number of MHC alleles that can be expressed per individual, which is expected to translate into a broader spectrum of recognized pathogens and higher fitness [26]. On the other hand, the expression of multiple MHC variants may carry some inherent costs related to resource allocation, autoimmune diseases or depletion of T cell receptor repertoire [27,28]. Because of these trade-offs, individual MHC diversity should be stabilized at certain (possibly fluctuating) species-specific evolutionary optima. These optima can be reached via gene duplication or pseudogenization/deletion events, generating substantial MHC gene copy number variation across species [29]. Owing to different pathogen-driven selective pressures, the evolutionary optima may also differ between MHC-I and MHC-II, promoting variations in gene copy numbers and overall genomic architecture between the two MHC classes [30].

 The MHC has long been recognized as the best system in vertebrates for investigating how natural selection promotes local adaptation at the genetic level [31]. To date, there is robust empirical support for local adaptation at MHC genes, although more evidence originates from research on MHC-II (e.g. [32,33]) than on MHC-I [34]. Direct comparisons of population differentiation between the two MHC classes suggest that local adaptation may indeed be stronger in MHC-II than in MHC-I [35,36]. This pattern may ultimately reflect stronger spatial variation in selection pressures imposed by extracellular pathogens compared with intracellular pathogens. In general, extracellular pathogens are taxonomically diverse, including not only microbes residing in the extracellular space but also multicellular macroparasites, such as helminths or ectoparasites [37,38]. Also, many macroparasites have complex life cycles with free-living stages or intermediate hosts, each with specific environmental and ecological requirements [39], which may create strong heterogeneity in the composition of parasite communities across landscapes and microhabitats. By contrast, intracellular parasites are generally more ‘internal’ to the host and, thus, less exposed to local variations in environmental conditions [40]. Additionally, intracellular parasites may spread more effectively over longer distances via their hosts [40], promoting spatial homogeneity of pathogen communities. Indeed, there is convincing evidence that many intracellular pathogenic viruses and bacteria show strong antigenic homogeneity throughout space [41]. Consequently, host population differentiation driven by local adaptation is expected to be stronger at MHC-II than MHC-I. However, despite this clear and simple prediction, robust comparative analyses of genetic differentiation between populations at both MHC classes across diverse host taxa are lacking.

The primary aim of this study was to test for differences in the extent of population differentiation between MHC-I and MHC-II. To this end, we compiled an extensive dataset of over 3500 pairwise estimates of population differentiation at the MHC across nearly 120 species from all major tetrapod lineages. We used a phylogenetically informed comparative approach to analyse these extensive empirical data and test for differences in population differentiation between the two MHC classes. In these analyses, we carefully not only controlled for variation in gene copy number and allelic diversity between MHC-I and MHC-II but also accounted for spatial population structure (geographical distances among populations), which affects the expected population differentiation by modulating the balance between drift and gene flow. Finally, to facilitate interpretation of the empirical results, we used simulations to investigate the effect of the genomic MHC architecture and its interaction with selection on the estimates of population differentiation.

## 2. Methods

### (a) Compilation of empirical data

 Data on population differentiation at the MHC genes across tetrapods were compiled from the literature. To this end, we initially conducted an advanced search in the Scopus database using the following query: TITLE-ABS-KEY (‘MHC’ AND ‘population*’) AND TITLE-ABS-KEY (‘differentiation’ OR ‘divergence’ OR ‘structure’ OR ‘diversity’) AND ALL (‘population differentiation’ OR ‘population divergence’ OR ‘population structure’ OR ‘genetic structure’). As this search retrieved numerous studies on domestic animals (e.g. cattle), laboratory animals (e.g. mice) and humans (including biomedical research), we refined the query by adding the following exclusion criterion: AND NOT TITLE-ABS-KEY (‘bovine’ OR ‘murine’ OR ‘patient*’ OR ‘human*’). The final search (conducted on 15 June 2024) yielded 464 published papers, predominantly addressing population differentiation in wild animal populations. The search strategy was validated against a set of relevant studies identified based on the authors’ prior knowledge of the literature, with the final search successfully retrieving all the benchmark studies. Of the retrieved records, 102 papers were excluded in the first filtering step as either being non-empirical (e.g. reviews or opinion pieces) or focusing on non-MHC genes or non-wild animals. Papers on fish (*n* = 87) were also excluded for three reasons: (i) many studies on fish were based on managed populations, in which patterns of population differentiation may not reflect natural processes; (ii) many fish taxa show extreme rearrangements of MHC architecture (e.g. complete loss of the MHC-II pathway [42,43]), potentially generating unique patterns of MHC gene flow and population differentiation; and (iii) population differentiation in fish and terrestrial tetrapods may be driven by distinct ecological factors, for example, reflecting different patterns of population (habitat) connectivity (e.g. gene flow in fish may be primarily modulated by waterway rather than straight-line geographical distance [44]). Taking all this into account, our study focused exclusively on four major lineages of tetrapods: amphibians, non-avian reptiles (henceforth: reptiles), birds and mammals. Hence, the remaining 275 papers were screened to extract information on MHC differentiation between populations across these lineages.

### (b) Major histocompatibility complex population differentiation

From the screened papers, we primarily extracted information on MHC population differentiation, as quantified using all available differentiation estimators, including *F*_ST_ and *F*'_ST_ (based on allele frequencies), *ϕ*_ST_ (based on molecular distance commonly applied to DNA sequence data), *G*_ST_, *G*′_ST_, *G*″_ST_, *K*_ST_, Rho and Jost's *D*. We also calculated MHC population differentiation from allele frequencies when such data were provided (e.g. in graphs or deposited raw datasets), but differentiation values were not reported directly in the text or tables (*n* = 31 papers). For this purpose, we used Jost's *D*, which is well suited for measuring the differentiation of allele frequencies when polymorphism within populations is high [45]. All calculations were performed using the *SpadeR* package [46] in R statistical environment.

 In total, we compiled 3734 pairwise MHC population differentiation values from 140 papers. However, the large majority (approx. 94%) of the dataset was dominated by only three differentiation estimators (*F*_ST_, *ϕ*_ST_ and Jost's *D*). These three complementary measures of population differentiation partly capture distinct aspects of genetic structure. *F*_ST_ quantifies differentiation as the proportion of total genetic variance attributable to differences among populations and is therefore sensitive to both allele-frequency differences and the amount of within-population variation. *ϕ*_ST_ is another variance-partitioning statistic, which additionally incorporates molecular distances among alleles or haplotypes, so that populations carrying more divergent sequences are considered more strongly differentiated than populations differing only in the frequencies of closely related variants. By contrast, Jost's *D* measures actual allelic differentiation by quantifying the degree to which populations contain distinct alleles, and is less constrained by high within-population diversity than is *F*_ST_. Estimators other than *F*_ST_, *ϕ*_ST_ and Jost's *D* were available for only a single study or taxon; therefore, to reduce methodological noise, these values were excluded from the dataset. We also excluded all pairwise estimates based on a total sampling effort of less than 10 individuals. After this filtering step, the final dataset comprised 3505 pairwise MHC population differentiation values from 130 papers. All these estimates were calculated using genotype data from exonic regions of MHC genes coding for the peptide-binding region (exon 2 and/or 3 for MHC-I and exon 2 for MHC-II). Because population differentiation values behave as proportion-like variables (usually bounded between 0 and 1), we performed a logit transformation prior to the analysis to remove boundaries and reduce right-skewness, producing an approximately normal distribution.

### (c) Species composition and phylogenetic relationships

The final dataset included 117 tetrapod species, but their representation was uneven among the four major lineages (figure 1). Mammals were the most strongly represented, accounting for over half (56%) of all species, whereas the lineages of amphibians and birds showed intermediate representation (20% each) and reptiles were poorly represented (only 4%) (figure 1). Although the distribution of estimates was clearly skewed towards MHC-II (approx. 85% of estimates), MHC-I was still represented by all major tetrapod lineages. Phylogenetic relationships among species were obtained from the complete time-calibrated tetrapod phylogenies available on the VertLife Web server [47–50]. To account for phylogenetic uncertainty, we used VertLife to download 1000 alternative phylogenetic trees for each tetrapod lineage and summarized them into consensus trees using Geneious v. 10.0.5 (Biomatters, Auckland, New Zealand). The four lineage-specific consensus trees were then manually merged into a single phylogeny based on the divergence times among the four major tetrapod lineages provided by VertLife.

**Figure 1.**
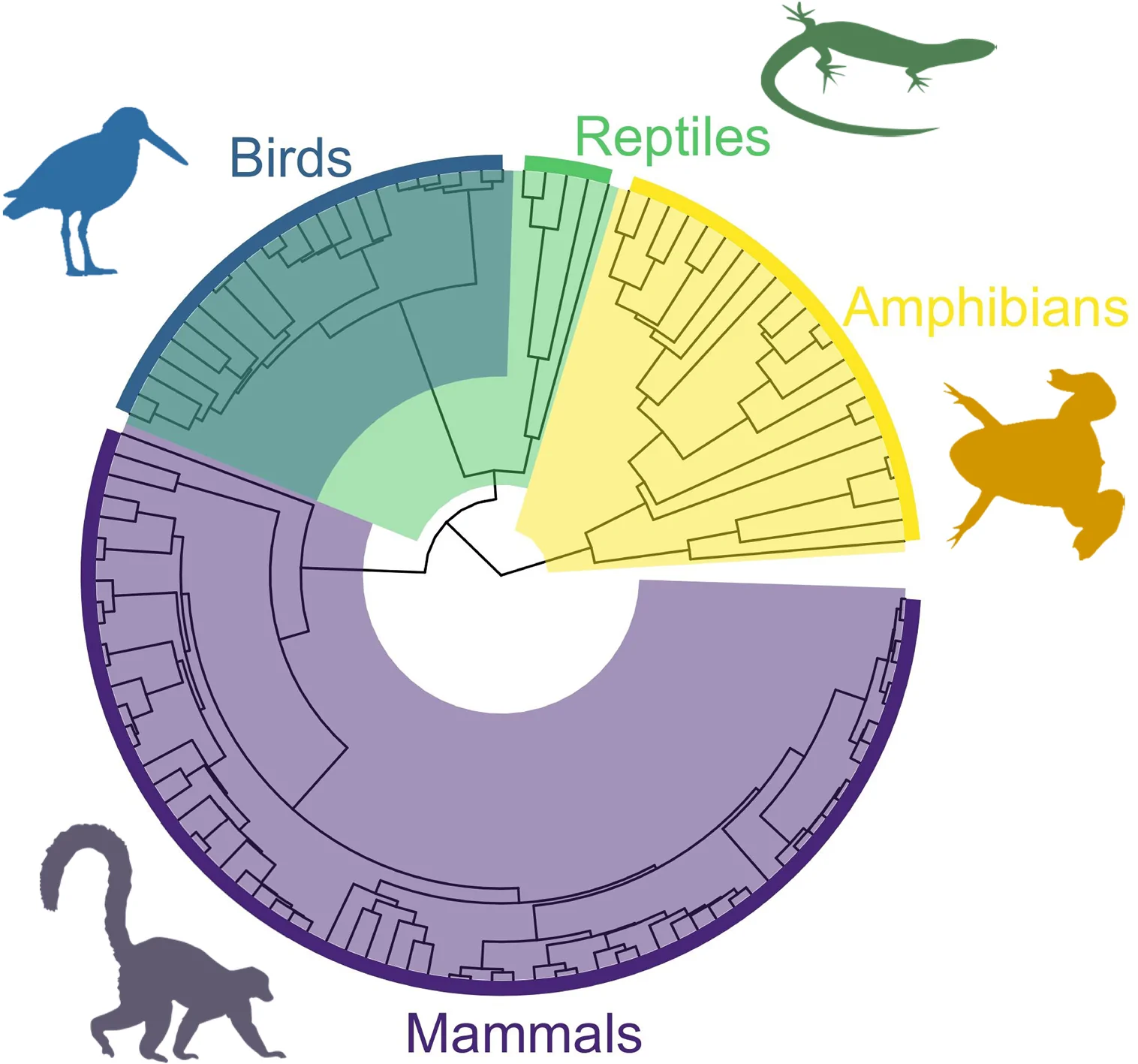
Phylogenetic distribution of species (*n* = 117) among four major tetrapod lineages used to investigate variation in population differentiation at the MHC genes.

### (d) Moderators

We compiled data on several moderators from the original sources. First, we collected information on MHC gene copy number and MHC allelic diversity for each study. In studies employing single-locus primers (e.g. divergent MHC genes in mammals), we recorded the exact number of genotyped loci (as reported in the original sources), whereas in studies using multi-locus primers (e.g. MHC genes homogenized by concerted evolution in birds), we inferred the number of genotyped loci by dividing the maximum number of alleles detected per individual by two, assuming heterozygosity at each locus. This conservative approach provides the minimum possible estimate of the number of loci per species. The number of genotyped MHC loci ranged from 1 to 13 per class, with most population differentiation estimates (62.7%) originating from only one or two loci per class. Estimates derived from intermediate (3) or high (≥4) numbers of loci were less frequent, accounting for 23.7 and 13.6% of the values, respectively. MHC allelic diversity was quantified as the total number of MHC allelic variants across all loci genotyped at a given MHC class and across all populations within each study (median: 24, range: 2–249 alleles per study). Pairwise geographical distances between populations were either obtained directly from the original sources (if reported) or inferred from the provided coordinates, study site names and maps. Overall, the dataset included MHC population differentiation estimates spanning a broad range of geographical scales (from 1 to approximately 13 000 km, median = 533 km). Finally, we compiled data on sampling effort, quantified as the number of individuals genotyped at the MHC per pairwise population comparison. A minimum threshold was set to 10 individuals (see above), while the maximum sampling effort was 801 individuals (median = 40 individuals). MHC allelic diversity, geographical distance and sampling effort were log_10_(*x* + 1)-transformed prior to analysis to reduce right-skewness and improve normality.

### (e) Phylogenetically informed comparative analyses

We used Bayesian general linear mixed models [51] to analyse the MHC population differentiation estimates across species. All models were run using the *MCMCglmm* R package [52]. In the models, we used MHC population differentiation as the response variable, while MHC class, MHC gene copy number, log MHC allelic diversity, log geographical distance and the type of estimator (*F*_ST_, *ϕ*_ST_ and Jost's *D*) were entered as predictors. The MHC gene copy number and log MHC allelic diversity were only moderately correlated in our dataset (Pearson product-moment correlation coefficient: *r* = 0.41) and thus were not expected to produce any multicollinearity issues. Study identity and species identity were entered as random factors in the model, whereas the non-independence of species due to shared evolutionary history was accounted for by an additional phylogenetically structured random effect (the animal term). The covariance matrix of this effect reflected phylogenetic relationships among species. The species identity effect therefore captured species-specific variation not explained by phylogeny, whereas the animal term captured the covariance among species arising from shared ancestry. To evaluate the effects of sampling effort, we applied two complementary approaches: (i) inclusion of sampling effort (log number of individuals) as an additional linear predictor, assuming it affected the expected value, but not the residual variance, and (ii) incorporation of sampling variance (inversely proportional to sampling effort) through the mev argument, treating it as an effort-based measurement error that affected the residual variance. Finally, the original phylogenetically informed *MCMCglmm* model was modified to assess differences in MHC population differentiation among major tetrapod lineages (included as a fixed effect). Since lineages were well aligned with the phylogeny (corresponding to deep phylogenetic splits) and were expected to capture substantial phylogenetic signals, the animal random effect linked to the phylogeny was removed from this model to avoid collinearity and interpretational ambiguity.

All *MCMCglmm* models assumed a Gaussian distribution of the response variable, consistent with the close-to-normal distribution of the logit-transformed differentiation values. In each model, we used uninformative inverse-Wishart priors (variance = 1, belief parameter = 0.002) for both R and G variance structures. Each model (chain) was run using 125 000 iterations with a burn-in of 25 000 and a thinning interval of 100, yielding an expected 1000 posterior samples. Consistent with this expectation, effective sample sizes for posterior means averaged 1042 ± 15 (s.e.) across models. Autocorrelation between successive samples was negligible (mean |*r*| = 0.023 ± 0.001 (s.e.) and |*r*| = 0.024 ± 0.001 (s.e.) for the mean and variance components, respectively). Convergence was assessed using two complementary approaches: (i) visual inspection of trace plots following Roy [53] and (ii) the Geweke [54] diagnostic, which evaluates differences in the means between the first 10% and last 50% of each chain using standardized *z*-scores (|*z*| < 1.96 used to infer adequate convergence).

### (f) Theoretical simulations

To investigate the effect of the genomic MHC architecture on the measures of MHC differentiation between populations, we performed individual-based forward simulations in SLiM4 [55], attempting to cover biologically realistic ranges of the relevant parameters. In each scenario, two populations, *N*_e_ = 500 individuals each, had evolved in isolation for 50*N*_e_ generations, then migration was switched on at a constant rate *m* (0.0001, 0.001, 0.01), and data were collected (30 individuals from each population) for *F*_ST_ calculation at times 0–4*N*_e_ generations following the start of migration, that is, until well after equilibrium should be attained under neutrality, even for the lowest migration rate [56]. We simulated two MHC classes evolving under NFS, with the additive effects of alleles on fitness. The strength of selection acting on each class was defined by selection coefficients *s*_MHCI_ and *s*_MHCII_ (0.1, 0.3 and 0.5), and we simulated scenarios where *s* was either equal or different for both classes. The values of *s* covered the range commonly reported in empirical MHC studies [25]. Selection was distributed equally among all loci of each class according to the multiplicative fitness model (see below). A total of 12 MHC loci were simulated in all scenarios, including 6, 9 or 11 loci at one MHC class (referred to as MHC-I) and the remaining loci (6, 3 or 1, respectively) assigned to the other class (referred to as MHC-II). Loci evolved under the infinite alleles model with per locus population mutation rate *θ* = 0.6, which corresponded to the mutation rate *µ* = 0.0003. Although this mutation rate is higher than what would typically be expected for MHC loci, it was scaled to the moderate effective population size used for computational tractability, thereby maintaining the intended and biologically plausible value of *θ*. Recombination between classes was *r*_inter (0.005, 0.5), corresponding to limited and free recombinations, respectively. Recombination between adjacent loci within each class, *r*_intra (0.0001, 0.001, 0.01), was set to reflect the range from extremely tight to moderate linkage. Thirty replicates were simulated for each scenario.

Fitness of an individual was determined as follows:

— for each class, take the number of loci (*n*_loc),
— calculate per-locus selection coefficient:
  $$\begin{array}{r} s\_loc=\;1-\left({\left(1-s\right)}^{1/n\_loc_{}}\right), \end{array}$$
— define fitness contribution of the locus *w_i_*:
  (i) if homozygous for an allele with population frequency *f*_1_, or one allele undefined (which may have occurred at the start of simulations), $w_{i}=\;1+\frac{s\_loc}{2}-f_{1}s\_loc$ and
  (ii) if heterozygous for alleles with population frequencies *f*_1_ and *f*_2_,
    $$\begin{array}{r} \;w_{i}=\frac{\left(1+\;\;s\_loc/2-f_{1}s\_loc\right)+\left(1+s\_loc/2-f_{2}s\_loc\right)}{2}, \end{array}$$
— calculate individual fitness by multiplying fitness contributions from all loci.

After sampling 30 individuals per population, genotypes were encoded as a binary allele presence/absence matrix; thus, each allele was effectively encoded as a separate dominant locus, and pairwise *F*_ST_ between populations was calculated using the *pairwise.fst.dosage* function from the *hierfstat* R package, which implements Weir & Goudet's [57] method.

## 3. Results

### (a) Comparison of population differentiation between the major histocompatibility complex classes—empirical results

Our general comparative model provided evidence of stronger population differentiation at the MHC-II than at MHC-I (*β* = 0.130, 95% CI: 0.039–0.216, *p* < 0.001; table 1 and figure 2) while controlling for several confounding variables, including the MHC gene copy number, MHC allelic diversity, geographical distance and the type of differentiation estimator. The MHC gene copy number was negatively associated with the strength of population differentiation, indicating weaker differentiation in species with more duplicated MHC genes (*β* = −0.040, 95% CI: −0.070 to −0.016, *p* = 0.004; table 1 and figure 3). By contrast, there was no association between population differentiation and the overall MHC allelic diversity (*p* = 0.708; table 1). As expected, MHC population differentiation increased with geographical distance (*β* = 0.194, 95% CI: 0.176–0.212, *p* < 0.001; table 1) and varied among the types of differentiation estimators, with *F*_ST_ and *ϕ*_ST_ values being significantly lower than Jost's *D* (table 1). The fixed effects explained a modest fraction of the variance (marginal *R*^2^ = 0.10), whereas the full model, including random effects, accounted for most of the variance (conditional *R*^2^ = 0.82). The results did not change qualitatively, while controlling for variation in sampling effort (supplementary material, table S1). Model testing for differences among major tetrapod lineages (instead of linking species to a phylogenetic tree) revealed significantly lower MHC population differentiation in birds (*β* = −0.504, 95% CI: −0.721 to −0.279, *p* = 0.002) and mammals (*β* = −0.388, 95% CI: −0.593 to −0.213, *p* < 0.001) than in amphibians (figure 4; supplementary material, table S2). Reptiles showed a similar level of population differentiation to amphibians (*β* = 0.100, 95% CI: −0.311 to 0.477, *p* = 0.630) (figure 4; supplementary material, table S2).

**Table 1.** The results of phylogenetically informed comparative Bayesian mixed models investigating variation in population differentiation at the major histocompatibility complex (MHC) genes across four major tetrapod lineages. Species and study identity were included as random factors; species were also linked to phylogeny through the animal term. Marginal and conditional *R*^2^ values are reported below the model results. Significant predictors are marked in bold.

predictor	estimate	lower 95% CI	upper 95% CI	*p*
**intercept**	**−0.671**	**−1.095**	**−0.245**	**0.002**
**MHC class (MHC-II versus MHC-I)**	**0.130**	**0.039**	**0.216**	**<0.001**
**MHC gene copy number**	**−0.040**	**−0.070**	**−0.016**	**0.004**
log MHC allelic diversity	**−**0.024	**−**0.146	0.101	0.708
**log geographical distance**	**0.194**	**0.176**	**0.212**	**<0.001**
**type of estimator (*F*_ST_ versus Jost's *D*)**	**−0.194**	**−0.247**	**−0.144**	**<0.001**
**type of estimator (*ϕ*_ST_ versus Jost's *D*)**	**−0.235**	**−0.347**	**−0.133**	**<0.001**
	marginal *R*^2^: 0.10		conditional *R*^2^: 0.82

**Figure 2.**
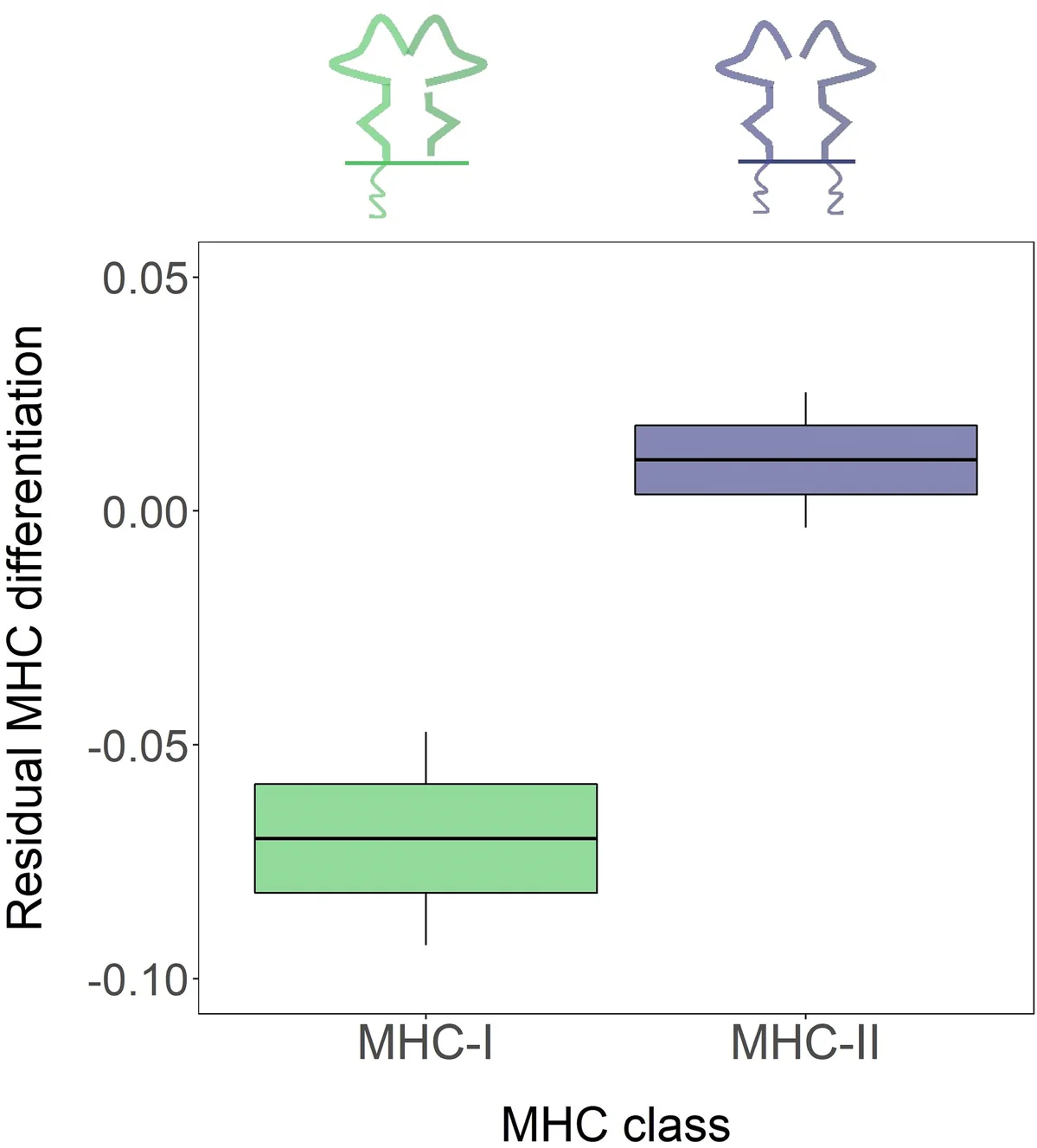
Variation in overall population differentiation between MHC-I and MHC-II. Central line—mean, box—s.e., whiskers—95% CI. Residual major histocompatibility complex (MHC) differentiation was extracted to remove variation from confounding variables (MHC gene copy number, log MHC allelic diversity, log geographical distance and type of estimator).

**Figure 3.**
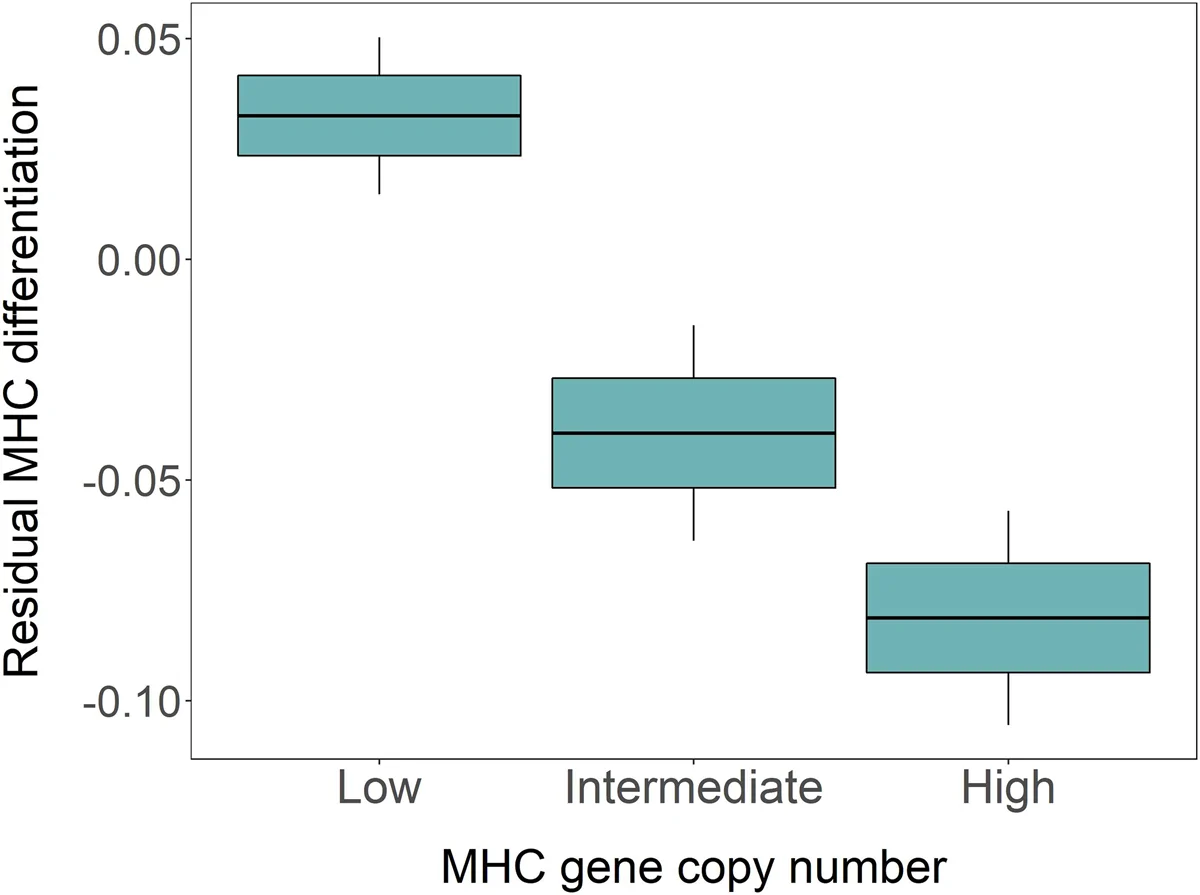
Variation in the overall population differentiation at the major histocompatibility complex (MHC) genes across species with different numbers of MHC gene copies. For the purpose of presentation, species were classified into three categories characterized by different numbers of MHC gene copies (low: 1–2 copies, intermediate: 3 copies, and high: ≥4 copies) . Central line—mean, box—s.e., whiskers—95% CI.

**Figure 4.**
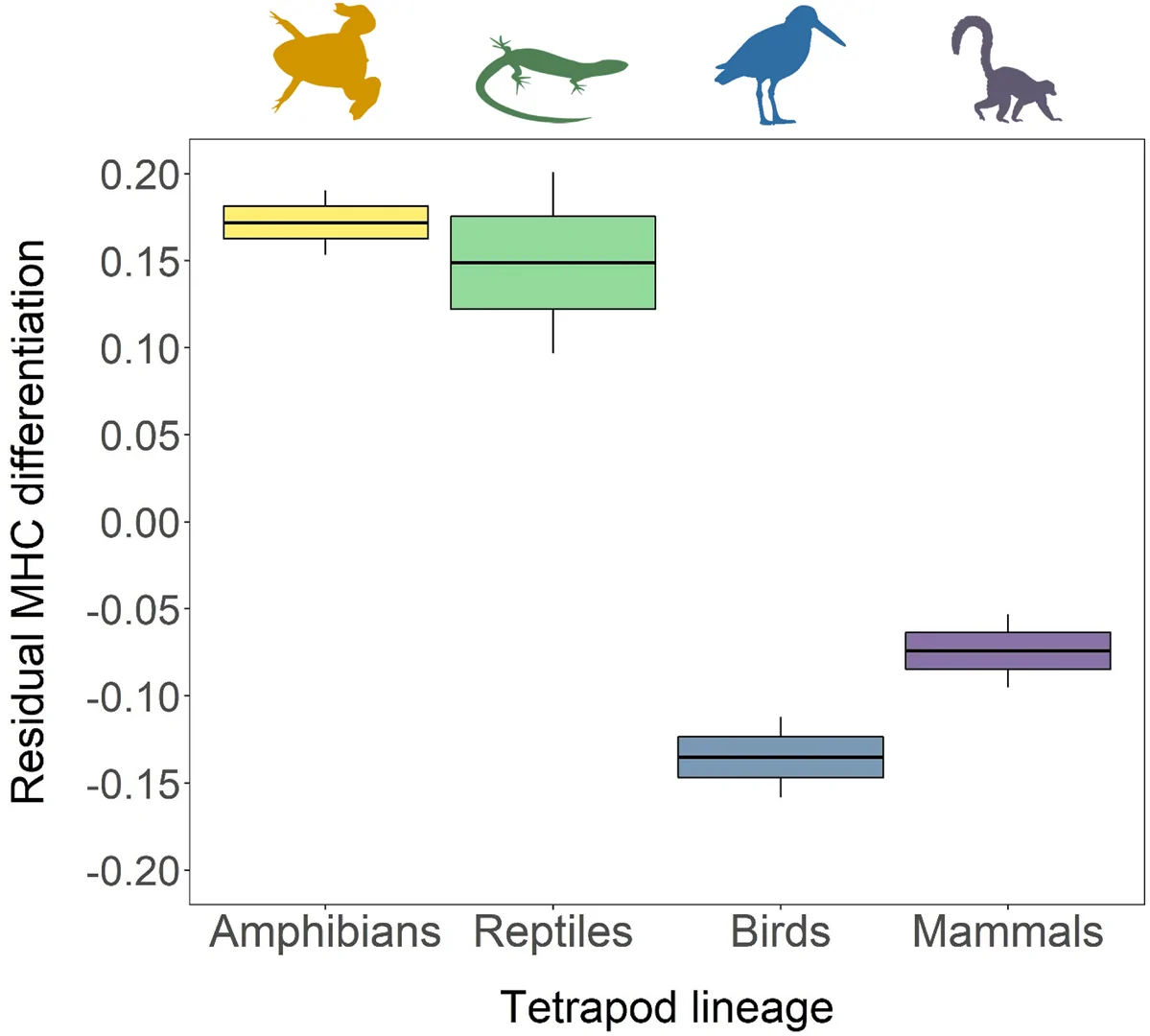
Variation in overall population differentiation at the major histocompatibility complex (MHC) genes among four major tetrapod lineages. Central line—mean, box—s.e., whiskers—95% CI.

### (b) Effects of the genomic major histocompatibility complex architecture on differentiation—simulation results

To facilitate interpretation of the empirical results, we explored how genomic MHC architecture (i.e. the number of genes per class) affects measures of genetic differentiation between populations. Specifically, we investigated whether differences in the number of genes between the MHC classes affect the level of differentiation under two contrasting scenarios, assuming either equal or different per-class strength of NFS. By contrast to our empirical results, simulations revealed that the level of population differentiation increased with the number of MHC genes when the per-class selection strength was equal. However, this pattern is reversed when the strength of selection differs between classes, such that the class with more loci shows lower *F*_ST_ when it is subject to a stronger NFS (figure 5; supplementary material, figure S1). These results suggest that the negative association of population differentiation with the number of MHC genes, as observed in the empirical data, may arise indirectly from the positive relationship between the number of loci and per-class selection strength. Our simulations also showed that the positive relationship between the number of loci and *F*_ST_ was strongest under weak selection and low migration, but it diminished over time since the onset of migration and may disappear entirely under strong selection combined with low intra-class recombination (figure 5; supplementary material, figures S1 and S2). Indeed, higher intra-class recombination amplifies this positive relationship (figure 5; supplementary material, figure S2), whereas recombination between classes has only a minor effect (supplementary material, figure S2).

**Figure 5.**
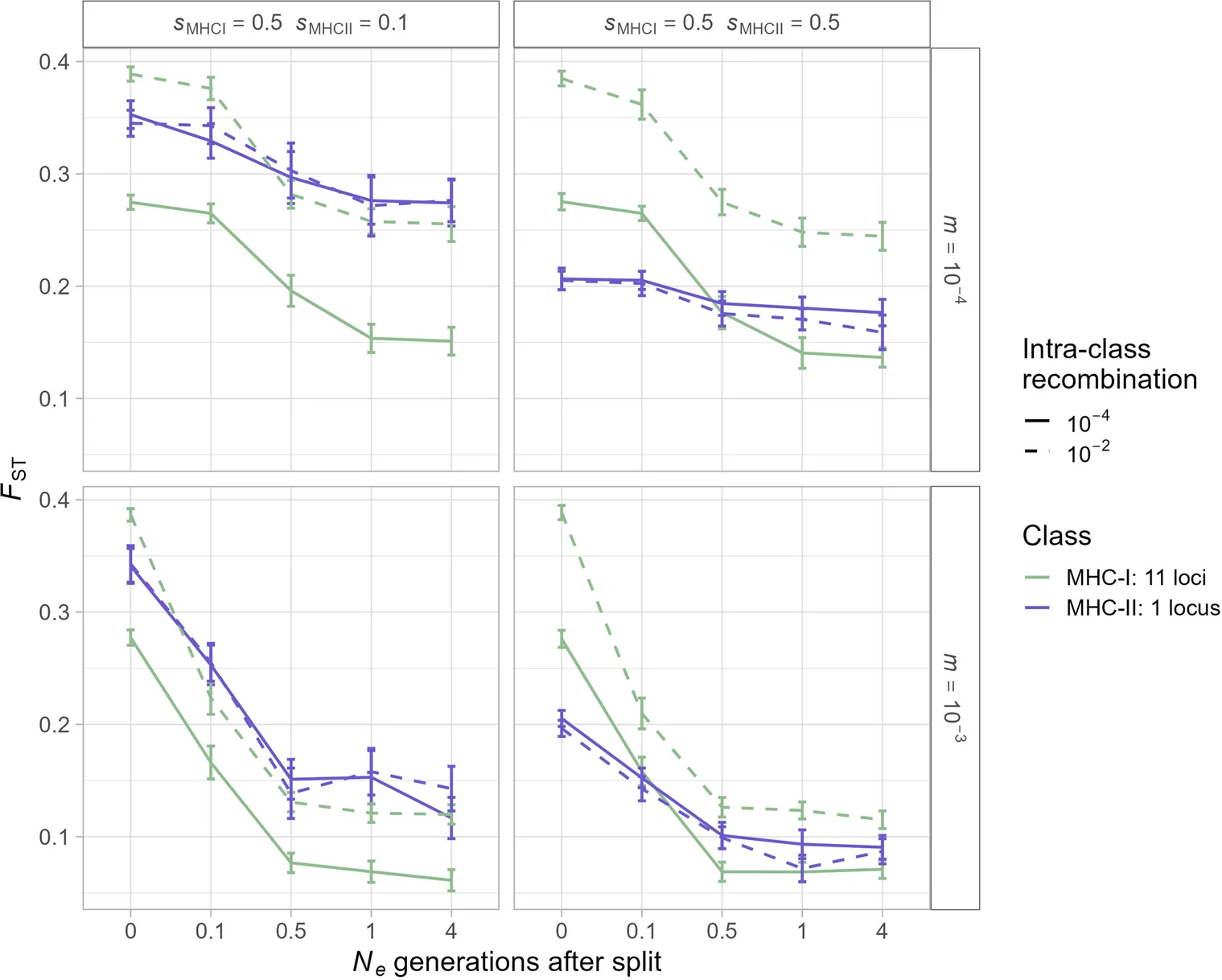
The results of simulations exploring the effect of the genomic major histocompatibility complex (MHC) architecture and selection strength on the MHC differentiation. Plots show pairwise *F*_ST_ between populations at different time points (measured in *N*_e_ generations) after the start of migration. Each plot shows the results for the difference of 11 loci versus 1 locus in the two MHC classes (labelled MHC-I and MHC-II, respectively). The left panels show the results for different per-class NFS strengths, whereas the right panels show the results for equal per-class selection strengths. The upper and lower panels show the results for two different migration rates (*m*). Lines connect means from 30 replicates, and error bars show the 95% CI. All scenarios assumed free recombination between MHC classes, whereas two different intra-class recombination rates are shown with different line types. Comparison of the left and right panels indicates that under equal per-class selection strength, a higher number of loci tend to elevate differentiation, while the effect is reversed if per-class selection is stronger for the class with more loci.

## 4. Discussion

In this study, we present robust comparative evidence for stronger population differentiation at MHC-II than at MHC-I genes across tetrapods. These results indicate that extracellular parasites may promote stronger local adaptation in genes involved in antigen presentation than intracellular parasites. We suggest that this pattern may be mediated by greater spatial variation in the composition of extracellular parasite communities and, consequently, stronger spatial heterogeneity in parasite-driven selection acting on MHC-II genes. At the same time, we provide empirical evidence that MHC differentiation among populations is modulated by the genomic architecture of this region, and we employ theoretical simulations to explore evolutionary scenarios linking MHC population differentiation to variation in gene copy number. Our analyses also revealed that isolation by distance contributes widely to the MHC differentiation, indicating that balancing selection cannot effectively homogenize MHC repertoire over large geographical scales. Finally, we demonstrate substantial variation in MHC differentiation among major host evolutionary lineages, broadly reflecting differences in dispersal capacity among tetrapods.

Stronger population differentiation at MHC-II than MHC-I has previously been reported in direct intraspecific comparisons. For example, MHC-II differentiation (mean *F*_ST_ = 0.40) was eight times stronger than MHC-I differentiation (mean *F*_ST_ = 0.05) in two species of Antarctic *Spheniscus* penguins [36]. A consistent pattern was observed across eight populations of the black-headed gull *Chroicocephalus ridibundus*, where the mean pairwise population differentiation (Jost's *D*) was estimated at 0.07 and 0.01 at MHC-II and MHC-I, respectively [35]. The same study showed that population differentiation at MHC-II, but not at MHC-I, was significantly stronger than differentiation at selectively neutral microsatellite markers, indicating that signatures of local adaptation were restricted to MHC-II [35]. Finally, MHC-II allelic variants showed much stronger (compared with MHC-I) associations with local habitat variation across three populations of the white-tailed eagle *Haliaeetus albicilla*, providing further support for stronger local adaptation at MHC-II [58]. Notably, most taxon-specific comparisons to date have been restricted to birds, which raised questions about the generality of this pattern. Here, we provide comprehensive and phylogenetically robust support for stronger population differentiation at MHC-II than at MHC-I genes across tetrapods. We argue that these results not only yield novel insights into the mechanisms shaping the diversity of these hyper-polymorphic immune genes but also advance our understanding of variation in parasite-driven selection regimes.

In particular, our findings suggest that the selection by extracellular parasites may exhibit stronger spatial variation across landscapes and habitats. First, extracellular parasites are likely to show greater differentiation in local community composition, reflecting their greater ecological diversification and more complex life cycles, including free-living life stages and intermediate hosts with specific ecological requirements [59]. Second, this taxonomic differentiation at the community level may be coupled with strong intraspecific antigenic differentiation of particular extracellular parasites across space, especially when species-specific levels of antigenic diversity are high. By contrast, the spatial differentiation of many intracellular parasites may be constrained by their remarkably low antigenic diversity [41]. Theoretical models attribute this low antigenic diversity to the high transmissibility of intracellular parasites (e.g. measles or rubella viruses), as high host contact rates, high transmissibility and short infection duration select for antigenic homogeneity [60]. Other (e.g. extracellular) parasites may trade transmissibility for the ability to generate multiple antigenically diverse strains in order to evade host immunity [60]. Taking all this into account, we suggest that stronger spatial antigenic differentiation of extracellular parasites is likely to drive stronger population differentiation at host MHC-II genes, as observed in this study.

While parasites are traditionally considered the ultimate factor shaping MHC variation, our analyses of empirical data and theoretical simulations indicate that the level of MHC differentiation is also modulated by MHC genomic architecture. Specifically, we found empirical evidence that a high gene copy number constrains MHC differentiation across populations. However, our simulations showed that under pure NFS, this pattern arose only when the per-class selection strength increased with the number of MHC loci. By contrast, when the per-class selection strength is equal for both classes, differentiation tends to increase with the number of loci. More broadly, our simulations highlight an important cautionary point: differences in *F*_ST_ between MHC classes can emerge even in the absence of local adaptation, driven instead by a complex interplay between the genomic MHC architecture, the strength of NFS and the duration of gene flow between populations. This underscores the need for careful statistical control of potential confounders in comparative analyses.

Our analysis identified isolation by distance as a general mechanism shaping MHC variation in tetrapods, indicating that MHC repertoires are not effectively homogenized across geographically distant populations. Isolation by distance emerges naturally under restricted gene flow and drift, but it can also be amplified by diversifying selection, promoting local adaptation, particularly when environmental conditions vary across space [8]. In this study, we were unable to determine whether selection contributed to isolation by distance at the MHC. Nevertheless, previous empirical research has provided ample support for the role of local adaptation in driving isolation by distance at the MHC. For instance, studies on the great snipe, *Gallinago media*, and house sparrow, *Passer domesticus*, revealed significant isolation by distance at the MHC while controlling for differentiation at neutral (microsatellite) markers [61,62]. However, some other species showed no evidence of isolation by distance at MHC genes [63,64]. Despite this taxon-specific variation, our study highlights the general importance of isolation by distance in driving MHC differentiation in tetrapods.

Finally, we found evidence of pronounced variation in the extent of geographical MHC structuring among major tetrapod lineages, with birds, mammals and amphibians/reptiles showing the lowest, intermediate and highest immunogenetic differentiation between populations, respectively. This pattern aligns well with the observed variation in dispersal capacity among taxonomic groups. Within tetrapods, both maximum dispersal distance and physiological vagility (i.e. speed at which an animal can move sustainably based on physiological parameters such as aerobic capacity, body temperature and metabolic costs of transport) are highest in flying birds and lowest in amphibians [65]. Consistently, the levels of neutral genetic differentiation, as quantified based on microsatellite markers, are lowest in birds and highest in amphibians, likely reflecting variation in dispersal rates [65]. Our findings indicate that species traits related to locomotion and dispersal are key predictors of gene flow among populations and, hence, determine the level of differentiation not only at neutral but also at adaptive genetic markers.

Although our empirical results were based on a large global dataset of MHC differentiation estimates, the analyses were not free from limitations. Most importantly, our data were unbalanced in terms of MHC class and tetrapod lineage representation, with most data compiled for MHC-II genes and mammals. Although it is difficult to formally assess whether and how this uneven data distribution affects the results, the high consistency of our general findings with previous taxon-specific studies supports the robustness of these patterns. However, our study identified clear gaps in the data, which merit specific recommendations for future studies on immunogenetic differentiation among populations. In particular, we recommend that research efforts should be redirected towards MHC-I genes (and possibly other immune genes involved in pathogen recognition that were not included in this study) and the most underrepresented taxonomic lineages, such as reptiles.

In conclusion, this study integrates extensive empirical data from over 100 tetrapod species with theoretical simulations to disentangle the mechanisms responsible for population differentiation at the most polymorphic vertebrate genes, those of the MHC. Our results advance the understanding of how extraordinary MHC variation is maintained across space and reveal global patterns of immunogenetic differentiation. Although we employed theoretical approaches to infer the evolutionary processes underlying the observed empirical patterns, we suggest that future research should also aim to explicitly link MHC variation with the taxonomic and antigenic diversity of pathogen communities. Because functional immunogenetic variation is recognized as an important predictor of species' adaptive potential [66], improved mechanistic understanding of host–parasite interactions and immune adaptation may have practical implications for the conservation management of fragmented populations, particularly under accelerating anthropogenic environmental changes that facilitate the spread of emerging diseases over large geographical scales.

## Supplementary Material



## Data Availability

The data and code supporting this manuscript have been archived in Zenodo repository [67]. Supplementary material is available online [68].
