# Supplementary material for: A global analysis of population differentiation at the major histocompatibility complex genes across tetrapods
Source: Proc Biol Sci. 2026 Oct 14;293(2080):20261096. doi: 10.1098/rspb.2026.1096 (PMC13678625; doi:10.1098/rspb.2026.1096)

Electronic Supplementary Material

A global analysis of population differentiation at the MHC genes across tetrapods

Piotr Minias[1], Amelia Chyb[1], Tomasz Gaczorek[2], Wiesław Babik[3]

[1]University of Lodz, Faculty of Biology and Environmental Protection, Department of Biodiversity Studies and Bioeducation, Banacha 1/3, 90-237, Lodz, Poland

[2]Jagiellonian University, Faculty of Biochemistry, Biophysics and Biotechnology, Laboratory of Bioinformatics and Genome Biology, Kraków, Poland

[3]Jagiellonian University, Faculty of Biology, Institute of Environmental Sciences, Kraków, Poland

Author for correspondence: Piotr Minias, e-mail: pminias@op.pl

DOI: 10.1098/rspb.2026.1096 (Proceedings of the Royal Society B)

Table S1 The results of phylogenetically informed comparative Bayesian mixed models investigating variation in population differentiation at the MHC genes across four major tetrapod lineages, while controlling for variation in sampling effort: A) sampling effort (log number of individuals) included as additional linear predictor, B) sampling variance included as effort-based measurement error in the model structure. Species and study identity were included as random factors, species was also linked to phylogeny through the animal term. Significant predictors are marked in bold.

Predictor	Estimate	Lower 95% CI	Upper 95% CI	p
**A. Intercept**	**-0.754**	**-1.185**	**-0.331**	**0.002**
**MHC class (MHC-II vs. MHC-I)**	**0.132**	**0.045**	**0.225**	**0.004**
**MHC gene copy number**	**-0.040**	**-0.070**	**-0.015**	**0.002**
Log MHC allelic diversity	-0.021	-0.143	0.098	0.722
**Log geographic distance**	**0.193**	**0.176**	**0.212**	**<0.001**
**Type of estimator (F_{ST} vs. Jost's D)**	**-0.195**	**-0.240**	**-0.144**	**<0.001**
**Type of estimator (φ_{ST} vs. Jost's D)**	**-0.236**	**-0.352**	**-0.138**	**<0.001**
Log number individuals	0.048	-0.010	0.109	0.134
**B. Intercept**	**-0.669**	**-1.092**	**-0.177**	**0.010**
**MHC class (MHC-II vs. MHC-I)**	**0.141**	**0.057**	**0.222**	**<0.001**
**MHC gene copy number**	**-0.043**	**-0.071**	**-0.014**	**0.008**
Log MHC allelic diversity	-0.010	-0.132	0.107	0.850
**Log geographic distance**	**0.194**	**0.175**	**0.210**	**<0.001**
**Type of estimator (F_{ST} vs. Jost's D)**	**-0.216**	**-0.264**	**-0.171**	**<0.001**
**Type of estimator (φ_{ST} vs. Jost's D)**	**-0.240**	**-0.349**	**-0.147**	**<0.001**

Table S2 The results of comparative Bayesian mixed models investigating variation in population differentiation at the MHC genes across four major tetrapod lineages. The tetrapod lineage was included as predictor instead of linking species to phylogeny through the animal term. Species and study identity were included as random factors. Marginal and conditional R^2 values are reported below the model results. Significant predictors are marked in bold.

Predictor	Estimate	Lower 95% CI	Upper 95% CI	p
**Intercept**	**-0.725**	**-1.045**	**-0.445**	**<0.001**
Tetrapod lineage (reptiles vs. amphibian)	0.100	-0.311	0.477	0.630
**Tetrapod lineage (birds vs. amphibian)**	**-0.504**	**-0.721**	**-0.279**	**0.002**
**Tetrapod lineage (mammals vs. amphibian)**	**-0.388**	**-0.593**	**-0.213**	**<0.001**
**MHC class (MHC-II vs. MHC-I)**	**0.134**	**0.040**	**0.219**	**0.002**
**MHC gene copy number**	**-0.042**	**-0.069**	**-0.016**	**0.004**
Log MHC allelic diversity	-0.010	-0.132	0.116	0.870
**Log geographic distance**	**0.193**	**0.175**	**0.211**	**<0.001**
**Type of estimator (F_{ST} vs. Jost's D)**	**-0.197**	**-0.248**	**-0.149**	**<0.001**
**Type of estimator (φ_{ST} vs. Jost's D)**	**-0.237**	**-0.346**	**-0.134**	**<0.001**
	Marginal R^2: 0.31		*Conditional R^2: 0.76*	

Figure S1 The results of simulations exploring the effect of the genomic MHC architecture on the genetic differentiation, highlighting the effects of the MHC loci number, per-class strength of negative frequency-dependent selection, and migration rate. Plots show pairwise F_{ST} between populations at different time-points (measured in the units of Ne generations) after the start of migration. Each plot shows results for 6 vs. 6, 9 vs. 3 and 11 vs. 1 loci in the two MHC classes (labelled MHC-I and MHC-II, respectively), as indicated with different line types. Panels show results for various combinations of the per-class strength of negative frequency-dependent selection (*s*, columns) and migration rate (*m*, rows). Lines connect means from thirty replicates and error bars show 95% confidence intervals. All scenarios assume free recombination between the MHC classes and intra-class recombination rate *r_intra* = 10^{-4}.

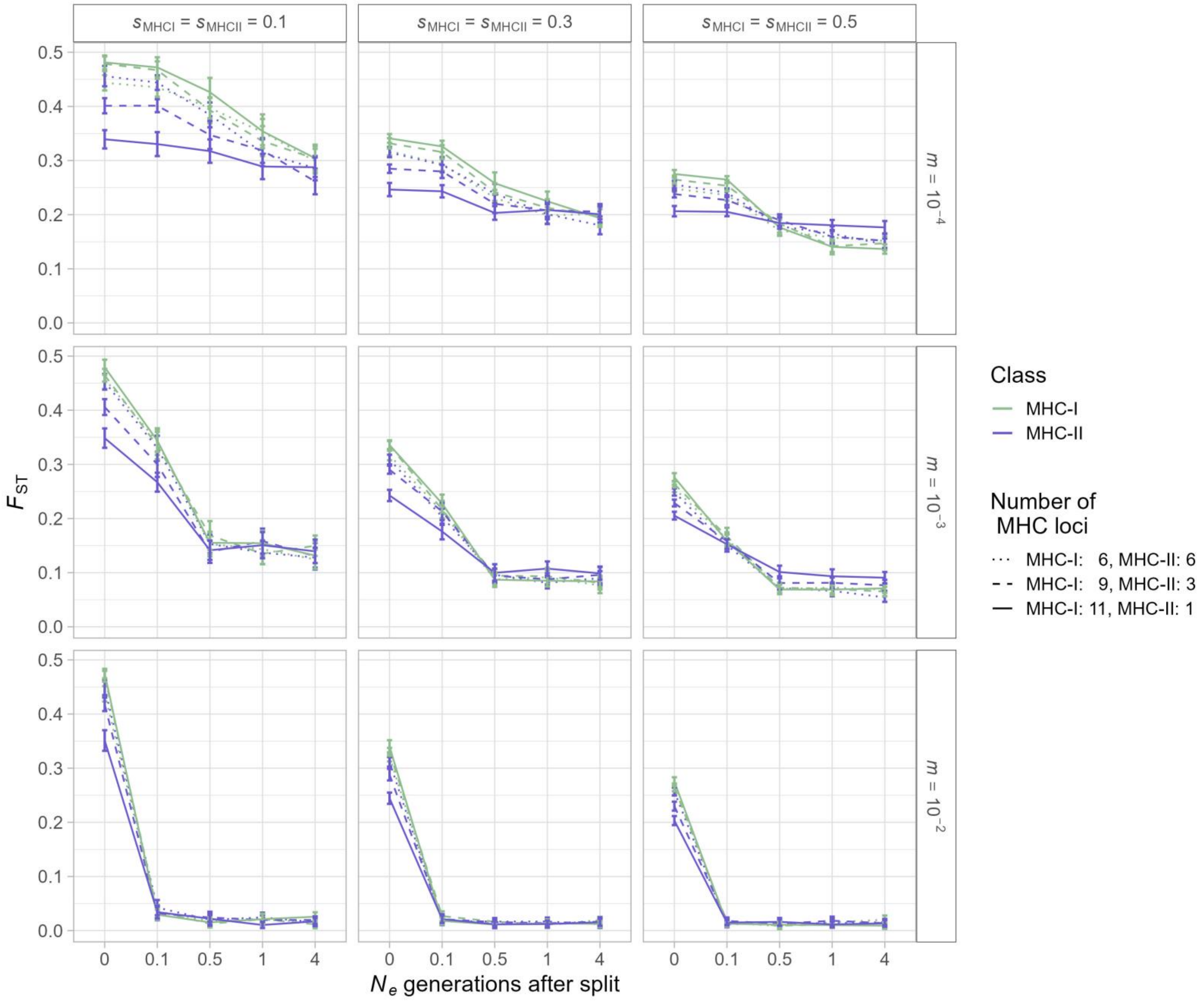

Figure S2 The results of simulations exploring the effect of the genomic MHC architecture on the genetic differentiation, highlighting the effects of the MHC loci number, between- and intra-class recombination rate, and migration rate. Pairwise F_{ST} between populations at different time-points (measured in the units of Ne generations) after the start of migration. Each plot shows results for different intra-class recombination rates (effectively only for multilocus MHC-I), as indicated with different line types. Panels show results for various combinations of the between-class recombination rate ($r_{\text{between classes}}$, columns) and migration rate (m, rows). Lines connect means from thirty replicates and error bars show 95% confidence intervals. All scenarios assume a difference of 11 loci vs. 1 locus in the two MHC classes (labelled MHC-I and MHC-II, respectively) and per-class strength of negative dependent selection s = 0.1.

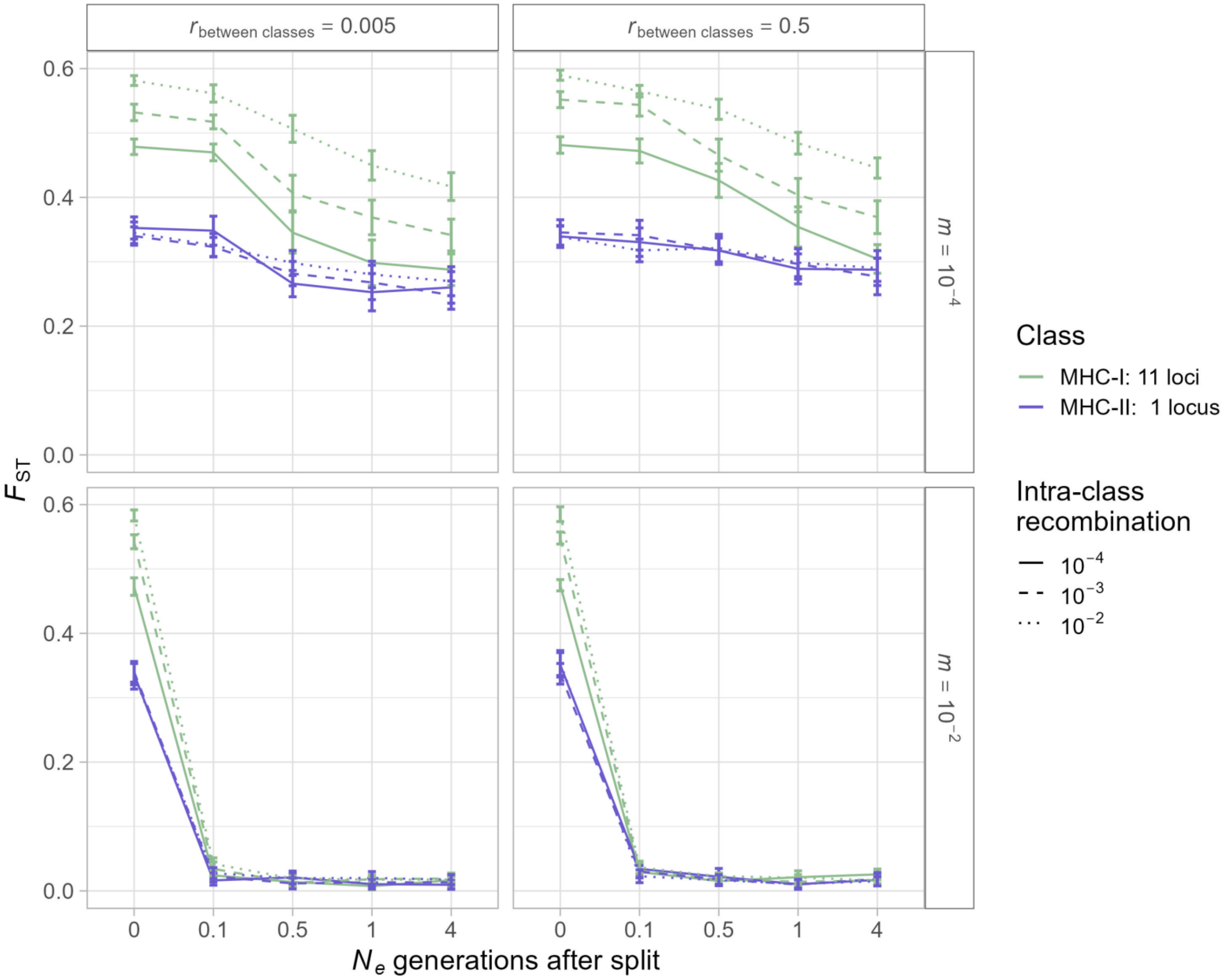

Supplement: Supplementary file 1 [file rspb.2026.1096.zip › rspb.2026.1096/rspb20261096_si_001.pdf]
